# Plasma Free Thiol Levels during Early Sepsis Predict Future Renal Function Decline

**DOI:** 10.3390/antiox11050800

**Published:** 2022-04-19

**Authors:** Elisabeth C. van der Slikke, Lisanne Boekhoud, Arno R. Bourgonje, Tycho J. Olgers, Jan C. ter Maaten, Robert H. Henning, Harry van Goor, Hjalmar R. Bouma

**Affiliations:** 1 Department of Clinical Pharmacy and Pharmacology, University of Groningen, University Medical Center Groningen, 9713 GZ Groningen, The Netherlands; e.c.van.der.slikke@umcg.nl (E.C.v.d.S.); l.boekhoud@umcg.nl (L.B.); r.h.henning@umcg.nl (R.H.H.); 2 Department of Gastroenterology and Hepatology, University of Groningen, University Medical Center Groningen, 9713 GZ Groningen, The Netherlands; a.r.bourgonje@umcg.nl (A.R.B.); h.van.goor@umcg.nl (H.v.G.); 3 Department of Internal Medicine, University of Groningen, University Medical Center Groningen, 9713 GZ Groningen, The Netherlands; t.j.olgers@umcg.nl (T.J.O.); j.c.ter.maaten@umcg.nl (J.C.t.M.)

**Keywords:** sepsis, acute kidney injury (AKI), reactive oxygen species (ROS), oxidative stress, free thiols

## Abstract

Sepsis is a life-threatening syndrome characterized by acute organ dysfunction due to infection. In particular, acute kidney injury (AKI) is common among patients with sepsis and is associated with increased mortality and morbidity. Oxidative stress is an important contributor to the pathogenesis of sepsis-related AKI. Plasma free thiols (R-SH) reflect systemic oxidative stress since they are readily oxidized by reactive species and thereby serve as antioxidants. Here, we aimed to assess the concentrations of serum free thiols in sepsis and associate these with major adverse kidney events (MAKE). Adult non-trauma patients who presented at the emergency department (ED) with a suspected infection were included. Free thiol levels and ischemia-modified albumin (IMA), a marker of oxidative stress, were measured in plasma at baseline, at the ward, and at three months, and one year after hospitalization. Plasma free thiol levels were lower at the ED visit and at the ward as compared to three months and one year after hospital admission (*p* < 0.01). On the contrary, plasma levels of IMA were higher at the ED and at the ward compared to three months and one year after hospital admission (*p* < 0.01). Furthermore, univariate logistic regression analyses showed that plasma free thiol levels at the ED were inversely associated with long-term renal function decline and survival at 90 days (MAKE90) and 365 days (MAKE365) (OR 0.43 per standard deviation [SD] [0.22–0.82, 95% CI], *p* = 0.011 and OR 0.58 per SD [0.34–0.96, 95% CI], *p* = 0.035, respectively). A multivariate regression analysis revealed an independent association of plasma free thiols at the ED (OR 0.52 per SD [0.29–0.93, 95% CI], *p* = 0.028) with MAKE365, even after adjustments for age, eGFR at the ED, SOFA score, and cardiovascular disease. These data indicate the clear role of oxidative stress in the pathogenesis of sepsis-AKI, as reflected in the lower plasma free thiol levels and increased levels of IMA.

## 1. Introduction

Sepsis is a complex, potentially life-threatening syndrome with organ failure due to a dysregulated host response to infection [1]. As such, sepsis is a major cause of death globally and one of the most common causes of death among hospitalized patients [1,2,3,4]. The precise mechanisms of sepsis-induced organ dysfunction are not fully understood, making it difficult to treat. As a result, current therapy is limited to source control and supportive care, including fluid resuscitation, antibiotic treatment, and, if necessary, organ support [4,5]. In particular, acute kidney injury (AKI) is common among patients with sepsis and is associated with increased mortality and morbidity [6,7]. Up to 60% of patients with sepsis develop AKI, and sepsis is the most common cause of AKI in the intensive care unit (ICU) [6,7]. Furthermore, there is increasing awareness that patients who survive sepsis often suffer from long-term morbidities and mortality, including chronic kidney disease (CKD) [1,4]. Both AKI and CKD lead to an increased long-term risk of end-stage renal failure and excess mortality [8,9].

Oxidative stress is an important contributor to the pathogenesis of sepsis-related kidney injury [10] and reflects the imbalance between reactive oxygen species (ROS) generation and antioxidant defense mechanisms [11,12]. Thiols are organosulfur compounds with a free sulfhydryl (-SH) group, which may act as electron acceptors and neutralize ROS by their oxidation. Serum albumin contains the largest amount of redox-active thiol groups, approximately 75% of the total thiol pool [13]. Its abundance makes thiols one of the most important protective mechanisms against oxidative stress in the extracellular environment by regulating redox exchange reactions and acting as potent antioxidants [14]. As such, oxidative stress can easily and reliably be monitored by measuring the plasma levels of reduced thiols, commonly indicated as ‘plasma free thiols’ [15,16]. Low plasma free thiols have already been associated with poor outcome in AKI and CKD, heart failure, and cancer [17,18,19,20,21]. However, the potential relationships between sepsis and plasma free thiol levels, as well as the long-term predictive capacity of free thiols with regard to future kidney injury, remain incompletely understood. In this study, we hypothesized that sepsis is associated with lowered levels of free thiols, mainly caused by hypoalbuminemia during sepsis, but also due to the scavenging of free radicals due to oxidative stress. We further hypothesized that lowered free thiols are associated with major adverse kidney events (MAKE). To address these hypotheses, we measured plasma free thiol levels at multiple time points in early sepsis and associated these with MAKE at 90 and 365 days after ED admission.

## 2. Methods

### 2.1. Study Population and Study Design

We conducted a prospective cohort study with a follow-up of two years. Adult non-trauma patients (>18–85 years of age) who presented at the emergency department (ED) of the University Medical Center Groningen (UMCG) with a suspected infection (as determined by the treating physician upon initial contact based on focal symptoms suggestive of an infection (e.g., productive cough, dyspnoea, dysuria, pollakisuria, abdominal pain, erythema)) and/or fever (≥38 °C, either at home or upon triage at the ED) which presented for internal medicine, rheumatology, or gastroenterology were screened for participation in this study. Next, patients who met two or more systemic inflammatory response syndrome (SIRS) criteria [22] (i.e., sepsis-2) were included. The SIRS criteria consisted of body temperature > 38 °C or <36 °C, heart frequency > 90 beats/min, respiratory rate > 20 breaths/min or PaCO_2_ < 32 mmHg, and white blood cell count > 12,000 cells/mm^3^, <4000 cells/mm^3^ or >10% immature (band) forms [22]. Written informed consent was obtained from all participating patients or given by proxy. The study protocol was approved by the medical ethical committee of the University of Groningen (in Dutch: “Medisch Ethische Toetsingscommissie”, METc, no. 2017/077). Patients were included from October 2017 to November 2019. The last moment of follow-up was November 2021.

### 2.2. Data Collection and Laboratory Measurements

Collected data included patient characteristics, demographic characteristics, vital function parameters, laboratory measurements, functional status, quality of life, and blood and urine samples. All data were gathered at hospital admission, on days 3–5 in hospital (referred to as the ‘ward’ time point), and three months and one year after hospital admission. Samples from two-year follow-ups were not used due to the small sample size. Blood and urine samples were immediately processed after collection and stored at −80 °C until further analysis. Mortality status was obtained from the Municipal Personal Records Database (BRP), containing reliable and complete registration of all Dutch citizens. Patient characteristics consisted of age, sex, race, and presence of comorbidities with diabetes mellitus, chronic kidney disease, cardiovascular disease, and cancer among others. Cardiovascular disease was defined as ischemic heart disease, heart failure, cardiac dysrhythmias, and peripheral arterial disease. Acute kidney injury (AKI) and chronic kidney disease (CKD) were defined according to the KDIGO criteria [23]. The Major Adverse Kidney Events (MAKE) criteria included mortality, new renal replacement therapy (RRT), or persistent renal dysfunction [24,25]. Vital parameters during hospital admission and days 3–5 consisted of heart frequency, systolic and diastolic blood pressure, mean arterial pressure, body temperature (tympanic), oxygen saturation, and respiratory rate. At three months and one year follow-up, heart frequency, systolic and diastolic blood pressure, mean arterial pressure, respiratory rate, and weight were collected. Laboratory measurements included leukocytes, thrombocytes, creatinine, albumin CRP, and eGFR. Other data included the source of infection, (q)SOFA score, medication use, length and height, family history, and length of hospital and intensive care unit (ICU) stay.

### 2.3. Post Hoc Sepsis Adjudication

Although patients were recruited based on a clinically suspected infection and the presence of at least two SIRS criteria, we performed a post hoc stratification of patients using sepsis-3 criteria. Sepsis-3 was defined as suspected or confirmed infection and the presence of two or more (quick) Sequential Organ Failure Assessment ([q] SOFA) criteria [1]. The SOFA score grades abnormality by organ system and includes the PaO_2_/FiO_2_ mmHg (kPa), platelet count, bilirubin level, Glasgow Coma Scale, MAP, catecholamine use, and creatinine level [1]. Severe sepsis was defined as sepsis with organ dysfunction, whereas septic shock was defined as persisting hypotension requiring vasopressors to maintain mean arterial pressure (MAP) ≥ 65 mmHg and having a serum lactate level > 2 mmol/L (18 mg/dL) despite adequate volume resuscitation [1].

### 2.4. Measurement of Plasma Free Thiol Levels

Plasma samples were stored at −80 °C until further analysis and thawed on ice overnight before use. Plasma free thiols were measured as previously described [26,27]. First, the samples were centrifugated at 10,000 rpm for 10 min at 4 °C. Next, 75-µL plasma was 4-fold diluted with 0.1-M Tris/EDTA buffer (pH 8.2), added to a flat-bottom 96-well plate in triplicates, and incubated for 20 min at room temperature. Next, 20 µL of 1 mM DTNB (5,5′-dithio-bis (2-nitrobenzoic acid) Ellman’s Reagent, Sigma Aldrich Corporation, St. Louis, MO, USA) in phosphate buffer (0.1 M, pH 7.0) was added, followed by incubation for 20 min at room temperature in complete darkness. Absorbance was measured at 630 nm (reference) and 412 nm (background absorption). A calibration curve with L-cysteine (15.625 µM to 1000 µM; Fluka Biochemika, Buchs, Switzerland) was made in 0.1-M Tris/EDTA buffer (pH 8.2). The total free thiol content was measured, consisting of the combination of protein-bound free thiols and low-molecular-weight (LMW) free thiols (e.g., cysteine, homocysteine, and glutathione) [13,16,28]. We both defined raw plasma free thiol concentrations and plasma free thiol concentrations corrected for albumin concentrations (presented in Supplementary Figures S1–S3). Plasma free thiol concentrations were corrected for plasma albumin by dividing free thiols through albumin concentrations since albumin is the most abundant human plasma protein and the predominant source of thiols [16].

### 2.5. Ischemia-Modified Albumin Measurement

Plasma ischemia-modified albumin (IMA) detection was assessed to define oxidative stress since IMA is considered a biomarker for oxidative stress and ischemia. Therefore, we used a rapid colorimetric method [29]. The method was modified using the principles of the IMA assay of the Szybio assay (Szybio Biotech, Wuhan, China) and Lee et al. [30]. Measurements were performed in duplo. Again, we both defined uncorrected IMA concentrations and IMA concentrations corrected for albumin concentrations in plasma (presented in the supplementary materials). To adjust for total albumin, the IMA/albumin ratio was used [31].

### 2.6. Statistical Analysis

Statistical analyses and data visualization were performed using R (RStudio Team: Integrated Development for R. RStudio, Inc., Boston, MA, USA) and SPSS 23.0.0.3 (IBM Corp. SPSS Statistics for Windows, Armonk, NY, USA). Descriptive statistics are presented as median and interquartile range (IQR), and proportions with corresponding percentages (n, %). Normal probability (Q-Q) plots and the Shapiro–Wilk test were used to test normality. Comparisons between different time points were performed using Wilcoxon’s matched-pair signed-rank tests. Baseline differences were tested using Mann–Whitney U tests. Correlations were tested with Spearman’s rank correlation coefficients (ρ). Univariable logistic regression analysis (pre-selection threshold: *p* < 0.10), followed by a multivariable logistic regression analysis was used to calculate the associations between important confounders in determining free thiol levels such as age, sex, SOFA score, plasma free thiols at hospital admission, diabetes mellitus, cardiovascular disease, eGFR, and malignancy with MAKE90 and MAKE365. MAKE90 is defined as having major adverse kidney events after 90 days and MAKE365 is defined as having major adverse kidney events after 365 days. A *p*-value of <0.05 was considered significantly different.

## 3. Results

### 3.1. Patient Characteristics

**Figure 1:**
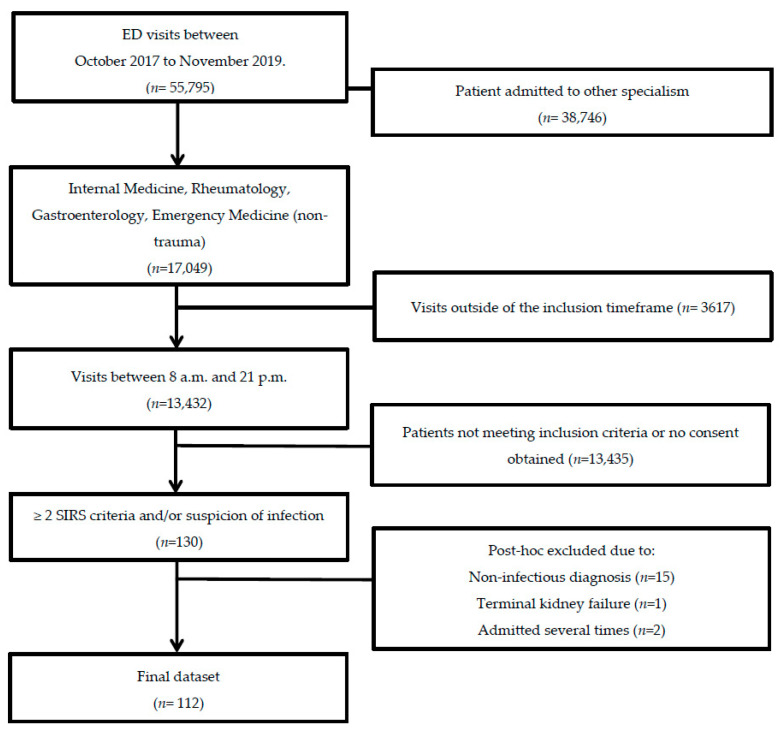
**Flow chart of patient selection.** Adult non-trauma patients who presented at the emergency department of the UMCG with a suspected infection between October 2017 and November 2019 were screened for inclusion.

In total, 143 patients were included in this study. We excluded 15 patients who did not have an infection, 13 patients due to a violation of inclusion criteria, and one patient due to pre-existent terminal kidney failure. When patients were admitted more than once during the study period, only the data from the first hospital admission were included in the analyses, resulting in a total study population of 112 patients (Figure 1). Baseline demographic and clinical data were obtained from 112 patients (Table 1). Of these 112 included patients, follow-up data from the ward were present for 83 (74%) patients, 58 (52%) patients at three months, and 39 (35%) patients after one year. The main reasons for loss-to-follow-up were patients who withdrew from study participation (*n* = 30, 27%), loss-to-follow-up without any particular reason (*n* = 13, 12%), or mortality (*n* = 27, 24%). There were two (2%) patients who died within seven days and 25 (22%) who died during follow-up. All patients met the sepsis-2 criteria and 66 patients (59%) met the sepsis-3 criteria. The median age at hospital admission was 65 (IQR: 16) years. AKI was present in 15 (13%) patients and CKD before hospital admission in 12 (11%). Patients were given antibiotics, fluid resuscitation, and supportive care. None of the patients received albumin transfusion.

### 3.2. Plasma Free Thiol Levels Are Lower during Sepsis

**Figure 2:**
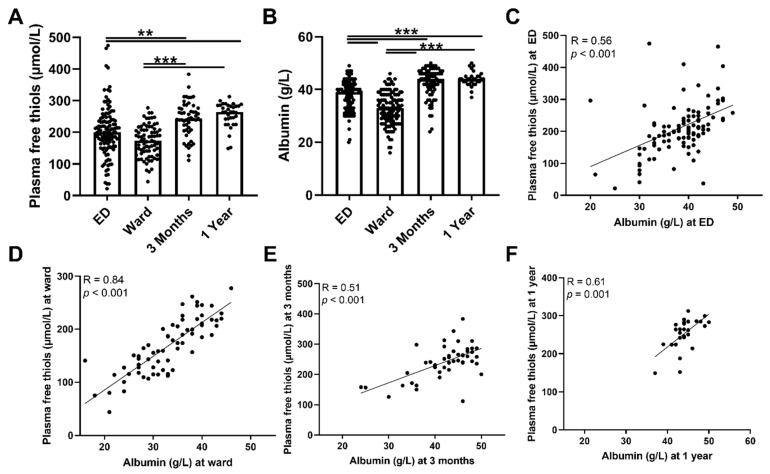
**Course of plasma free thiols in patients with sepsis and correlations with albumin.** (**A**) Plasma free thiols at the ED, the ward, and at three months and one year after hospital admission. (**B**) Albumin levels at the ED, the ward, and at three months and one year after hospital admission. (**C**) Correlation of plasma albumin at the ED and plasma free thiols at the ED. (**D**) Correlation of plasma albumin at the ward and plasma free thiols at the ward. (**E**) Correlation of plasma albumin at three months and plasma free thiols at three months. (**F**) Correlation of plasma albumin at one year and plasma free thiols at one year. Statistical analyses were performed using Wilcoxon matched-pairs signed test. Bars represent the median; dots represent individual levels. ** means *p* < 0.01 and, *** *p* < 0.001. ED: emergency department.

To assess antioxidant capacity, oxidative stress, and their course, plasma free thiol- and ischemia-modified albumin levels were measured during admission to the ED and at follow-up. Plasma free thiol levels at the ED and the ward were not significantly different, but both were lower compared with three months and one year after hospital admission (Figure 2A, both *p* < 0.01). To assess whether a depletion of albumin was accompanied by a decrease in plasma free thiols, we correlated plasma free thiols with albumin, which constitutes the most abundant carrier of plasma free thiols. Albumin levels and plasma free thiols corrected for albumin were significantly lower at the ED and the ward compared to the three-month and one-year levels (Figure 2B and Supplementary Figure S1, respectively). In addition, ED albumin levels correlated with ED plasma free thiols (Figure 2C, Spearman’s Rho = 0.56, *p* < 0.001), as did they at the ward (Figure 2D, Spearman’s Rho = 0.84, *p* < 0.001), at three months follow-up (Figure 2E, Spearman’s Rho = 0.51 *p* < 0.001), and at one year follow-up (Figure 2F, Spearman’s Rho = 0.61, *p* = 0.001). Thus, albumin depletion in sepsis was correlated with a decrease in plasma free thiol levels. Furthermore, higher plasma free thiol levels at baseline correlated with higher plasma free thiols at three months and at one year.

### 3.3. Plasma Free Thiols Are Associated with Ischemia-Modified Albumin

**Figure 3:**
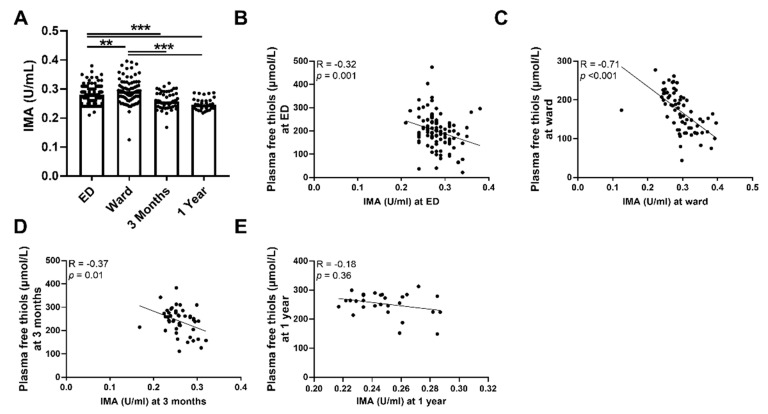
**Course of IMA in septic patients and association with plasma free thiols.** (**A**) IMA concentrations at the ED, the ward, and at three months and one year after hospital admission. (**B**) Correlation of IMA at the ED and plasma free t ian the ED. (**C**) Correlation of IMA at the ward and plasma free thiols at the ward. (**D**) Correlation of IMA at three months and plasma free thiols at three months. (**E**) Correlation of IMA at one year and plasma free thiols at one year. Statistical analyses were performed using Wilcoxon matched-pairs signed test. Bars represent the median; dots represent individual levels. ** means *p* < 0.01, *** *p* <0.001. ED: emergency department, IMA: ischemia-modified albumin.

To further sustain our initial findings of higher levels of systemic oxidative stress as reflected by plasma free thiol levels, we performed an additional measurement of IMA. Indeed, we observed that the plasma levels of IMA were higher during the ED visit and at the ward compared to three months and one year after hospital admission (Figure 3A, *p* < 0.001). This was also the case after albumin correction (Supplementary Figure S2). Plasma levels of IMA were inversely correlated with plasma free thiol levels at all time points (Figure 3B–D). IMA levels at the ED negatively correlated with albumin levels at the ED (Spearman’s Rho = −0.70, *p* < 0.001), IMA levels at the ward negatively correlated with albumin levels at the ward (Spearman’s Rho = −0.86, *p* < 0.001), and IMA levels at three months negatively correlated with albumin levels at three months (Spearman’s Rho = −0.58, *p* < 0.001). IMA levels at one year did not correlate with plasma free thiol levels at one year (Figure 3E). Furthermore, IMA levels at the ED positively correlated with CRP (Spearman’s Rho = 0.35, *p* < 0.001), age (Spearman’s Rho = 0.2, *p* = 0.04), SOFA score (Spearman’s Rho = 0.28, *p* = 0.004), the amount of oxygen suppletion at the ED (Spearman’s Rho = 0.29, *p* = 0.008), and length of hospital stay (Spearman’s Rho = 0.27, *p* = 0.006), but negatively correlated with oxygen saturation at the ED (Spearman’s Rho = −0.365, *p* = 0.012). Thus, sepsis is accompanied by higher levels of systemic oxidative stress as reflected in the lower plasma free thiol levels and higher levels of IMA during the ED visit and at the ward, compared to three months and one year after hospital admission.

### 3.4. Lower Plasma Free Thiols Are Associated with Renal Function Decline

**Figure 4:**
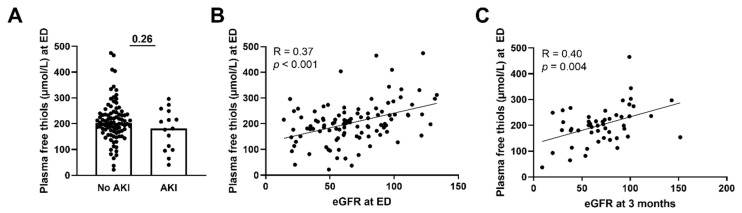
**Correlation of plasma free thiols with kidney function.** (**A**) Plasma free thiol levels at the ED in patients with and without AKI. (**B**) Correlation of plasma free thiols and eGFR at the ED. (**C**) Correlation of plasma free thiols and eGFR at three months. Statistical analysis in panel A was performed using a Mann–Whitney U test. Bars represent the median; dots represent individual levels. AKI: acute kidney injury ED: emergency department eGFR: estimated glomerular filtration rate.

Next, we assessed whether plasma free thiols were associated with several disease parameters and the presence of AKI. Plasma free thiol levels at the ED of patients with sepsis-induced AKI were similar to those without AKI (Figure 4A, *p* = 0.26). This was also the case for the albumin-adjusted plasma free thiols (Supplementary Figure S3A). However, plasma free thiols at the ED positively correlated with the eGFR at the ED (Figure 4B, Spearman’s Rho = 0.37, *p* < 0.001). Additionally, plasma free thiols at the ED negatively correlated with age (Spearman’s Rho = −0.25, *p* = 0.01), CRP (Spearman’s Rho = −0.29, *p* = 0.003), creatinine (Spearman’s Rho = −0.33, *p* = 0.001), the amount of oxygen suppletion (Spearman’s Rho = −0.34, *p* = 0.002), SOFA score (Spearman’s Rho = −0.26, *p* = 0.007), and the length of the hospital stay (Spearman’s Rho = −0.42, *p* < 0.001). Furthermore, we examined the correlation between plasma free thiols and their effect on kidney function during long-term follow-up. Here we observed that plasma free thiols at the ED were negatively correlated with serum creatinine at three months (Spearman’s Rho = −0.32, *p* = 0.024) but positively correlated with the eGFR at three months (Figure 4C, Spearman’s Rho = 0.40, *p* = 0.004). Furthermore, albumin-adjusted plasma free thiols at the ED were positively correlated with the eGFR at the ED and at three months (Supplementary Figure S3B,C).

### 3.5. Plasma Free Thiols Are Associated with Long-Term Renal Function Decline

To study whether plasma free thiol levels were associated with long-term renal function decline and mortality we associated plasma free thiol levels with MAKE90 and MAKE365, composite measures including mortality, new renal replacement therapy (RRT), or persistent renal dysfunction at 90- and 365-days follow-up, respectively. Univariate logistic regression analysis showed that plasma free thiols at the ED (OR 0.43 per standard deviation [SD] [0.22–0.82, 95% CI], *p* = 0.011), eGFR at the ED (OR 0.52 per SD [0.96–0.99, 95% BI], *p* = 0.028), and SOFA score (OR 1.47 [1.13–1.91, 95% CI], *p* = 0.004) were associated with MAKE90. In contrast, age, diabetes mellitus, cardiovascular disease, and malignancy were not significantly associated with MAKE90. A multivariate logistic regression analysis revealed an independent association of the SOFA score with MAKE90 (OR 1.41 [1.07–1.85, 95% CI], *p* = 0.014) persisting after adjusting for cardiovascular disease, eGFR, and plasma free thiols (Table 2). Both eGFR and plasma free thiols at the ED were not significantly independently associated with MAKE90.

In addition, plasma free thiols at the ED (OR 0.58 per SD [0.34–0.96, 95% CI], *p* = 0.035), SOFA score (OR 1.28 [1.03–1.60, 95% CI], *p* = 0.028), age per 10 years (OR 1.40 [1.00–1.96, 95% CI], *p* =0.047), and malignancy (OR 4.0 [1.61–9.96, 95% CI], *p* = 0.003) were univariately associated with MAKE365. In contrast, eGFR at the ED, diabetes mellitus, and cardiovascular disease were not associated with MAKE365. A multivariate logistic regression analysis showed an independent association of plasma free thiols at the ED (OR 0.52 per SD [0.29–0.93, 95% CI], *p* = 0.028) and malignancy (OR 4.4 [1.66–11.58, 95% CI], *p* = 0.003) with MAKE365, even after adjustment for age, eGFR at the ED, SOFA score, and cardiovascular disease (Table 2). Thus, plasma free thiols, eGFR at the ED, and SOFA score were associated with MAKE90, where the SOFA score emerged as an independent predictor of MAKE90. Additionally, plasma free thiols at the ED, SOFA score, and malignancy were associated with MAKE365, where both plasma free thiol levels at the ED and malignancy appeared to be independent predictors of MAKE365.

## 4. Discussion

This study demonstrates that baseline plasma free thiols upon ED admission can predict long-term renal function loss and survival after one-year follow-up in patients with sepsis, independent of the SOFA score, eGFR, malignancy, cardiovascular disease, and age. Furthermore, we showed that plasma free thiols were lower among patients with early sepsis upon visiting the ED and at the ward compared to after three months and one year after hospital admission, indicating that oxidative stress levels were higher at the ED and the ward compared to three months and one year after hospital admission. In contrast, IMA levels were higher during the ED visit and at the ward compared to three months and one year after hospital admission, denoting high oxidative stress levels. Additionally, albumin depletion in sepsis correlated with lower plasma free thiol levels, emphasizing the potential importance of albumin as an antioxidant. Finally, plasma free thiol levels upon ED admission positively correlated with the eGFR at the ED and three months after hospital admission.

Oxidative stress is an important contributor to the development of organ damage in sepsis [12]. It can be measured as a depletion of free thiol levels in plasma, which constitute the main determinants of the whole-body redox status [14,15,16]. With less buffering of reactive species by plasma free thiols, the excessive production of ROS causes significant cellular dysfunction in organs, which contributes to the inability to treat sepsis as well as multi-organ failure [32]. Many patients with sepsis develop AKI, and sepsis survivors often suffer from chronic kidney disease (CKD) [1,4,6,7]. We did not find any differences in the plasma free thiol levels in septic patients with or without AKI. However, the lack of a statistically significant difference in plasma free thiols is most probably due to the small sample size and thus a type 2 error, since only 13% of the patients suffered from AKI. Recently, lower levels of plasma free thiols have already been associated with poor disease outcome in AKI and CKD [17,21]; however, the course of free thiol levels during sepsis and their associations with major adverse kidney endpoints was not yet uncovered. Our study is the first to explore the relationship between plasma free thiol levels in patients with sepsis and renal function impairment in time. It confirms the presence of oxidative stress in sepsis [12], as shown by initially decreased plasma free thiol levels and increased IMA levels, compared to three months and one year after hospital admission. Oxidative stress is an important contributor to the pathogenesis of sepsis-related kidney injury [10]. The importance of antioxidants in surviving sepsis is illustrated by a higher antioxidant potential during the first two weeks of hospitalization for survivors of sepsis as compared to non-survivors [33]. Several thiol-based antioxidants, such as glutathione (GSH) and thioredoxin, have been studied for their potential to restore extracellular thiol homeostasis in both human and animals [34,35,36]. Administration of these substances as a potential therapeutic treatment seems promising for averting organ damage in sepsis, as it reduces oxidative stress, improves the acute physiology and chronic health evaluation II (APACHE II) score, and improves the logistic organ dysfunction (LOD) score in septic patients and strongly enhances survival in septic mice [35,37]. Thus, plasma free thiols could serve as a biomarker for monitoring the disease course in early sepsis while it is still potentially amenable to therapeutic interventions.

The inverse correlation between plasma free thiol levels and renal function during hospital admission is in line with our previous findings in patients with sepsis in the ICU where plasma free thiols were negatively correlated with plasma creatinine [21]. The predictive value of plasma free thiols for renal function decline among sepsis survivors after hospital admission was not yet known. However, post-operative plasma free thiol levels in kidney graft recipients are associated with eGFR after one year [38]. Univariate logistic regression analyses showed that plasma free thiol levels at the ED were inversely associated with long-term renal function decline and survival at 90 days (MAKE90) and 365 days (MAKE365), suggesting that high plasma free thiols are protective against renal function decline after hospital admission. The association between lower plasma free thiols at the ED and long-term outcome is consistent with previous findings, demonstrating a correlation between low plasma free thiols and all-cause mortality in the general population [19,39]. Hence, the inverse association between plasma free thiols and long-term kidney function and mortality emphasizes its potentially added value as a biomarker in predicting long-term mortality after sepsis.

Albumin levels in sepsis were correlated with plasma free thiol levels upon ED admission, which suggests that the observed thiol depletion may be at least partially attributed to lower levels of albumin. Sepsis, and especially sepsis-AKI, is often associated with hypoalbuminemia as a result of decreased synthesis, increased utilization by tissues, and increased transcapillary leakage from blood vessels due to increased vascular permeability [40]. In our cohort of patients with early sepsis at the ED, however, albumin levels were in the lower normal range (20–49 g/dL). Albumin can be regarded as an integrative biomarker of redox-sensitive events since the single free thiol (-SH) group of circulating albumin has a high redox-active potential. In addition, it is the major contributor to extracellular free thiol levels, since it not only contains the largest amount of redox-active thiol groups with approximately 75% of the total thiol pool, but also because of its transporting capacity of low molecular weight (LMW) thiols [13]. As such, a depletion of albumin and a decrease in free thiol levels marks a state of high oxidative stress [16]. If albumin supplementation in patients could restore thiol levels with a consequent beneficial effect in patients with sepsis, is not yet demonstrated [41]. However, one meta-analysis showed that the administration of albumin-containing solutions was associated with lower mortality compared with crystalloids [42]. Also, albumin infusion improves renal function in non-septic patients with liver cirrhosis with AKI by improving renal blood flow autoregulation [43] and reducing oxidative stress-related AKI [44]. Lastly, in patients with sepsis, albumin oxidation levels are inversely associated with 90-day mortality rate, independent of shock or sepsis treatment and a higher pool of reduced albumin correlated with a lower SOFA score at dayseven [45]. Thus, human plasma albumin is an important circulating antioxidant and holds promise for the treatment of sepsis.

The strengths of our study include its prospective design, with the collection of data and blood from patients with early sepsis directly upon triage at ED admission and at long-term follow-up. Potential limitations, however, constitute its single-center design in a tertiary care hospital with a referral of patients for academic specialist care, which may limit external generalizability. Yet our hospital has a substantial geographical spread in a rural area, ensuring a diverse population in need of both academic and non-academic care. Another limitation is that we used the sepsis-2 criteria to include patients upon ED admission, of which only 60% met the sepsis-3 criteria. However, this may have resulted in an underestimation of the effect of sepsis on plasma free thiol and IMA levels, since the sepsis-3 criteria includes patients with more severe sepsis compared to the sepsis-2 criteria. Indeed, we found lower plasma free thiol levels upon ED admission in patients meeting the sepsis-3 criteria compared with those meeting the sepsis-2 criteria. Furthermore, we did not include any control subjects in our cohort, yet we do know from our previous work that plasma free thiols in controls are significantly higher compared to patients with oxidative stress-mediated diseases such as inflammatory bowel disease, diabetes, and COVID-19 [20,46,47,48]. As sepsis is known for its extremely high ROS production [11,12], we assume that the free thiol levels are higher in control subjects compared to septic patients. This is supported by the fact that the plasma free thiol levels in patients with type 2 diabetes were almost three times as high when compared to our septic patient cohort [47], and patients with COVID-19 had 30% higher plasma free thiol levels compared to septic patients [48]. Lastly, it is unlikely that plasma free thiols would be fully representative of the global extracellular redox state as more redox-related substances are present. However, within the total antioxidant capacity, systemic free thiols lie at the center of the reactive species interactome (RSI), and they act as the major scavengers of reactive species in addition to the relatively minor antioxidant contributions of, for example, bilirubin and uric acid. As they provide a robust monitoring tool for translational studies, they have been analyzed as an integrative biomarker of systemic oxidative stress in a variety of oxidative-stress-mediated conditions before, e.g., renal disease, cardiovascular disease, diabetes mellitus, and inflammatory bowel disease [17,19,20,49]. Other antioxidant compounds such as glutathione, homocysteine, or cysteine, comprise low-molecular-weight (LMW) thiols, and are of minor importance to the antioxidant capacity, as the greatest share is represented by the protein free thiols (60–75%) [13]. In addition, it is not always the case that they accurately represent the overall redox state or are part of a central redox hub, and instead reflect spill-over products originating from upstream inflammatory processes [50]. Thus far, existing knowledge is insufficient to define the relative contributions of all these individual compounds and their specific roles in redox signaling pathways. In light of these considerations, the single quantification of serum free thiols is currently considered one of the most useful, high-throughput screening tools for measuring the whole-body redox status in translational settings.

## 5. Conclusions

In this study we showed that plasma free thiols were lower during the ED visit and at the ward, compared to three months and one year after hospital admission in patients with early sepsis, indicating high levels of oxidative stress during early sepsis. Also, plasma free thiols at the ED were independently associated with kidney injury-free survival (major adverse kidney endpoints, MAKE) at 365 days. Plasma IMA levels, a marker of oxidative stress, were inversely correlated with plasma free thiol levels. Finally, albumin depletion in sepsis was strongly correlated with a decrease in plasma free thiols at the ED, suggesting that thiol depletion could at least be partially attributed to lower albumin levels. These data indicate the clear role of oxidative stress in the pathogenesis of sepsis-AKI, as reflected by the lower plasma free thiol levels and increased levels of IMA. Future studies may include the thiols as therapeutic targets to improve sepsis care.

## Figures and Tables

**Table 1:** Baseline study population characteristics.

	All Patients *n* = 112
Age (years)	66 (17)
Gender, males	67 (60%)
Diabetes mellitus Malignancy Cardiovascular disease Chronic Kidney Failure	28 (25%) 48 (43%) 42 (38%) 12 (11%)
Thrombocytes (×10^9^ L)	209 (129)
CRP (mg/L)	81 (112)
Leukocytes (×10^9^ L)	9.7 (8.6)
Creatinine (µmol/L)	97 (49)
Albumin (g/L)	39 (8)
eGFR mL/min/1.73 m^2^	63 (41)
SIRS > 2 (sepsis-2)	112 (100%)
SOFA score ≥ 2 (sepsis-3)	66 (59%)
AKI AKI 1 AKI 2 AKI 3	15 (13%) 13 (12%) 1 (1%) 1 (1%)
ICU admission	8 (7%)

All laboratory measurements were obtained upon admission to the ED. Data are shown as the median with interquartile range or *n* with percentage. Cardiovascular disease included: ischemic heart disease, heart failure, cardiac dysrhythmias, and peripheral arterial disease. SIRS: systemic inflammatory response syndrome, AKI: acute kidney injury, eGFR: estimated glomerular filtration rate, ICU: intensive care unit.

**Table 2:** Uni- and multivariate logistic regression analysis with composite endpoints MAKE90 and MAKE365.

FACTORS	MAKE 90				MAKE 365			
	Univariate		Multivariate		Univariate		Multivariate	
	OR (95%CI)	*p* Value	OR (95% CI)	*p* Value	OR (95%CI)	*p* Value	OR (95% CI)	*p* Value
Plasma free thiols at ED (per SD)	0.43 (0.22–0.82)	0.011			0.58 (0.34–0.96)	0.035	0.52 (0.29–0.93)	0.028
SOFA Score (per point)	1.47 (1.13–1.91)	0.004	1.41 (1.07–1.85)	0.014	1.28 (1.03–1.61)	0.028		
eGFR at ED (per SD)	0.52 (0.96–0.99)	0.028			0.66 (0.97–1.00)	0.071		
Age per 10 years	1.23 (9.86–1.78)	0.28			1.40 (1.00–1.96)	0.047		
Malignancy	2.14 (0.75–6.1)	0.16			4.0 (1.61–9.96)	0.003	4.40 (1.66–11.58)	0.003

Model characteristics multivariate regression analysis: MAKE90 R^2^ = 0.06, *n* = 105, chi-square = 6.2, *p* = 0.013, and MAKE365 R^2^ = 0.131, *n* = 105, chi-square = 14.69, *p* = 0.001. Abbreviations: CI, confidence interval; OR, odds ratio.

## Data Availability

Data is contained within the article and Supplementary Material.

## Supplementary Materials

The following supporting information can be downloaded at: https://www.mdpi.com/article/10.3390/antiox11050800/s1, Figure S1: Course of albumin adjusted plasma free thiols; Figure S2: Course of albumin adjusted IMA in septic patients; Figure S3: Correlation of albumin adjusted plasma free thiols with kidney function.
