# Supplementary material for: Plasma Free Thiol Levels during Early Sepsis Predict Future Renal Function Decline
Source: Antioxidants (Basel). 2022 Apr 19;11(5):800. doi: 10.3390/antiox11050800 (PMC9137477; doi:10.3390/antiox11050800)
Supplement: Supplementary file 1 [file antioxidants-11-00800-s001.zip › antioxidants-1676053-supplementary.pdf]

Supplemental

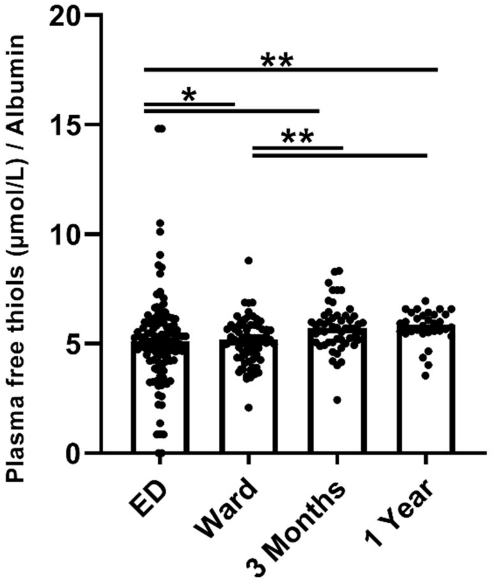

Figure S1. Course of albumin adjusted plasma free thiols. Albumin adjusted plasma free thiols at the emergency department, at the ward, and 3 months and 1 year after hospital discharge. Statistical analysis was performed using Wilcoxon matched-pairs signed test. Bars represent the median, dots represent individual levels. * means $p < 0.05$, ** $p < 0.01$. ED; emergency department.

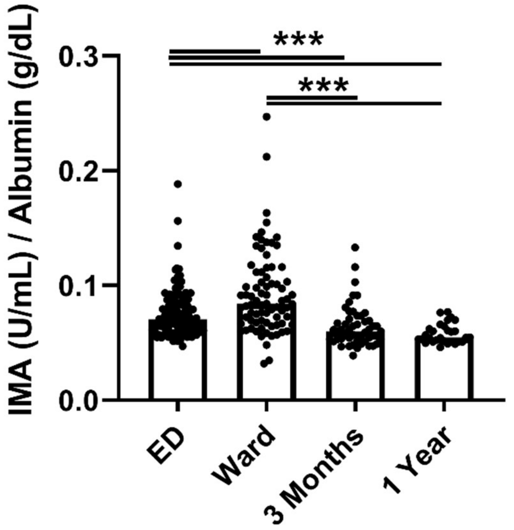

Figure S2. Course of albumin adjusted IMA in septic patients. IMA concentrations at the ED, the ward, at 3 months and one year after hospital admission. Statistical analysis were performed using Wilcoxon matched-pairs signed test. Bars represent the median, dots represent individual levels. *** means $p < 0.001$. ED: emergency department, IMA: ischemia-modified albumin.

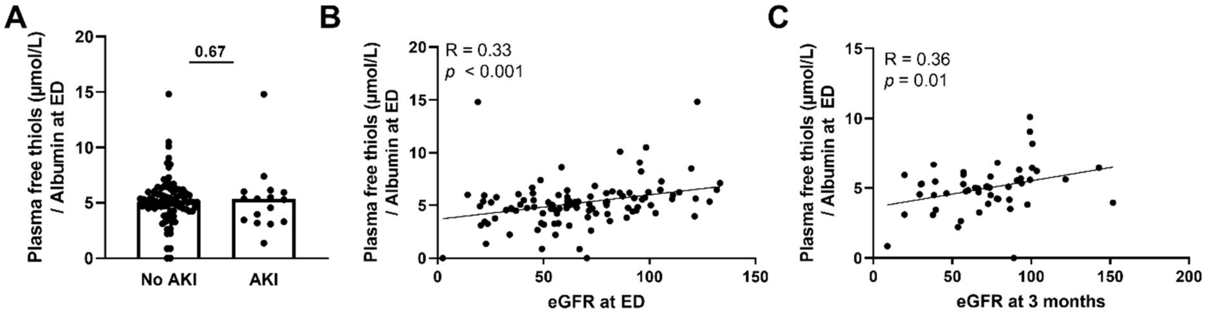

Figure S3. Correlation of albumin adjusted plasma free thiols with kidney function. (**A**) albumin adjusted plasma free thiols in patients with and without AKI. (**B**) Correlation of albumin adjusted plasma free thiols at ED and eGFR at ED. (**C**) Correlation of albumin adjusted plasma free thiols at ED and eGFR at three months. Statistical analysis were performed using Mann Whitney u test. Bars represent the median, dots represent individual levels. ED: emergency department, eGFR: estimated glomerular filtration rate.
